# In Vitro Vascular Network Modified to Function as Culture Platform and Angiogenic Induction Potential Test for Cancer Cells

**DOI:** 10.3390/ijms21051833

**Published:** 2020-03-06

**Authors:** Outi Huttala, Synnöve Staff, Tuula Heinonen, Johanna Mäenpää, Minna Tanner, Timo Ylikomi

**Affiliations:** 1 Cell Biology, Faculty of Medicine and Health Technology, Tampere University, FI-33014 Tampere, Finland; 2 FICAM, Faculty of Medicine and Health Technology, Tampere University, FI-33014 Tampere, Finland; 3 Tays Cancer Center, Tampere University Hospital, FI-33520 Tampere, Finland; 4 Department of Obstetrics and Gynecology, Tampere University Hospital, FI-33520 Tampere, Finland; 5 Faculty of Medicine and Health Technology, Tampere University, FI-33014 Tampere, Finland; 6 Department of Oncology, Tampere University Hospital, FI-33520 Tampere, Finland; 7 Department of Oncology, Faculty of Medicine and Health Technology, Tampere University, FI-33014 Tampere, Finland

**Keywords:** angiogenesis, in vitro assay, human adipose stromal cells, patient-derived cancer cells, personalized medicine, functional assay, patient-specific, cancer, human umbilical vein endothelial cells

## Abstract

Drug treatments have been designed to inhibit tumor angiogenesis in hope of stopping tumor growth. However, not all tumor types respond to this type of treatment. A screening method which identifies angiogenesis inducing cancer types would help predict the efficacy of angiogenesis-inhibiting drugs for the patients. Our goal is to develop (1) a cell assay to assess the angiogenic induction potential of patient-derived tumor cells, and (2) a protocol for culturing cancer cells on a vascular platform. We optimized the media composition and seeding density of cells (hASC, HUVEC, and cancer cells) to 48-, 96-, and even 384-well plate sizes to allow vascular formation and cancer cell proliferation and subsequent analysis with high throughput. The angiogenic induction potential of patient-derived cancer cells was investigated by quantifying the formation of tubular structures and the drug response of cancer cells grown on a vascular platform was evaluated using gene expression and cell viability (WST-1) assay. Immunocytochemistry was performed with von Willebrand factor, collagen IV, CD44, cytokeratin 19 and ALDH1A1. The angiogenic induction potential test was shown to be responsive to the induction of angiogenesis by cancer cells. The responses of cancer cells were different when grown on a vascular platform or on plastic, seen in gene expression level and viability results. These two protocols are promising novel tools for aiding the selection of efficient cancer drugs for personalized medicine and as an alternative cancer cell culture platform.

## 1. Introduction

Blood supply is essential for tumor growth and progression. It is needed to ensure the supply of nutrients and oxygen, but also to remove waste products from the tumor. In tumor-induced angiogenesis, tumor cells secrete angiogenic factors into surrounding normal tissue, which, in turn, promotes the blood vessel formation into the tumor tissue. Due to the need of blood vessels, one effective means to prevent growth and expansion of tumors is the inhibition of angiogenesis. Angiogenesis-inhibiting drugs are targeting various key phases of the angiogenesis process, including angiogenesis inducing growth factors such as VEGF, VEGF pathway, matrix metalloproteases, and αvβ3-integrin–vitronectin interaction [1].

The growing number of cancer drugs on the market is making it more difficult to choose the right drug for each patient. Biomarker and gene expression profile studies have not been able to predict the treatment efficacy sufficiently. Thus, patient-derived cancer cell cultures have been used to test the efficacy of the drugs. To date, this has been carried out by using cell culture methods based on the culture of cancer cells on cell culture plastic. These plastic 2D cultures fail to recapitulate the environment of in vivo tumors as they lack the correct cell-to-cell interactions, extracellular matrix composition, and structure of in vivo tissue, among others [2,3]. The traditional 2D method, although easy and scalable to high throughput, may provide false results, negative and positive, as shown in several studies [2,3,4,5,6]. Thus, more accurate and predictive systems are needed.

The culture of primary cancer cells, especially cancers such as prostate, is difficult to keep alive. To overcome the viability issues of patient-derived cancer cells, patient-derived xenografts grown in immunodeficient mice have been utilized. However, the animal biology-based culture environment poorly predicts the human outcome and treatment efficiency [7]. Most promising growth environments have been constructed in 3D as the cellular responses to drug treatments in 3D cultures have been shown to be more similar to what occurs in vivo compared to 2D culture [8,9,10]. However, 3D cultures often require culture times not suitable for fast patient sample screening, where answers are needed quickly.

We have previously developed a human in vitro angiogenesis assay that produces network of in vitro vasculature structures. The assay consists of human adipose stromal cell (hASC) and human umbilical cord vein endothelial cell (HUVEC) co-culture and forms 3D tubule structures similar to in vivo vasculature [11]. The vasculature, which forms in the assay, has been shown to be a beneficial platform for multiple cell types, including cardiomyocytes [12] and adipocytes [13]. Due to the criticalness of crosstalk between cancer cells and their microenvironment, especially with cells forming the vasculature, we hypothesize that the traditional 2D culture can be greatly improved by adding a vascular component to the culture. The main goals of this study are to modify the angiogenesis assay (1) to test the angiogenic induction potential of cancer cells, and (2) to function as a culture platform for cancer cells as an option for traditional protocols where cancer cells are grown on plastic.

## 2. Results

### 2.1. Scaling of Vascular Structures

In order to implement the developed cell assays in high throughput studies, the cell numbers were optimized for 48-, 96-, and 384-well plate formats. The cell densities of hASC and HUVEC (20000 and 4100 cells/cm^2^, respectively) were based on the previously published angiogenesis model, which has been intra-laboratory validated according to OECD guidance [14]. The optimization of cancer cell culturing was performed in preliminary work (not included in the article). The optimization study showed that most optimal cell numbers for cancer cells were 10000 cells per 48-well plate, 5000 cells per 96-well plate, and 1000 cells per 384-well plate. These cell numbers allowed enough room for cells to proliferate during the culture period and there were enough cancer cells compared to the number of vasculature cells, i.e., hASC and HUVEC. The 384-well plate used had square-shaped wells compared to the 48- and 96-well plates. The cells in square wells tend to prefer growing in the middle of the well, and hence the optimal cell number/cm^2^ is smaller than in the 96-well plate. On the 48-well plate, 10,000 cancer cells occupied the whole well area evenly. This cell number was the highest possible, the cells detached from the well before day 7 when more than 10,000 cancer cells were plated. This was most likely due to an overgrowth of cells resulting in a lack of nutrients. It must be borne in mind that the vascular platform is alive with metabolically active dividing cells.

**Figure 1:**
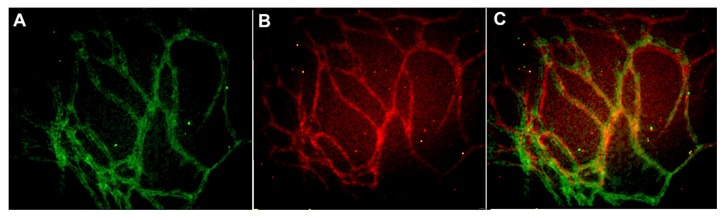
Vascular structures cultured on 384-well plate and stained with (**A**) anti-collagen IV (FITC-green) and (**B**) anti-von Willebrand factor (VWF) (TRITC-red) antibodies. The merged image is seen in (**C**). The edges and square shape of the 384 well plate caused some challenges in obtaining representative images from the formed vascular network. Image obtained with Cell-IQ with 10x objective.

The scaling of vascular structures to 48-well, 96-well, and 384-well plate sizes was successful in terms of formed continuous endothelial tubule network, visualized by von Willebrand factor (VWF) and intact basement membrane visualized by collagen IV (Figure 1). Hence the system is scalable to high throughput formats.

### 2.2. Characterization of Vascularized Cancer Assay

The vascularized cancer assay protocol developed in this study was compared to the traditional culture protocol of cancer cells grown on plastic. Commonly used cancer cell lines, including PC3, LNCAP, and MCF7, were utilized. The co-culture of cancer cells and vasculature (named as vascularized cancer assay) was assessed for both the existence of proper vasculature and the viability of cancer cells. Vascular structures were stained with anti-VWF for analyzing the endothelial layer and with anti-collagen IV for analyzing the basement membrane. Cancer cells were stained with cytokeratin 19 (CK19), CD44, and ALDH1A1. CK19 is a marker that has been shown to be expressed in MCF7 cells [15], and here it is used to show that the phenotype of MCF7 is correct and to study the localization of MCF7 cell compared to vasculature. CD44 is a surface protein involved in cell–cell interactions, cell adhesion, and migration [16]. ALDH1A1 is widely used to characterize the stemness of cancer cells and is also connected to cell migration and tumor metastasis (https://www.ncbi.nlm.nih.gov/pmc/articles/PMC5653849/). CD44 and ALDH1A1 were studied to see whether the vascular platform influences the expression of these markers in MCF7 compared to the expression reported in the literature.

**Figure 2:**
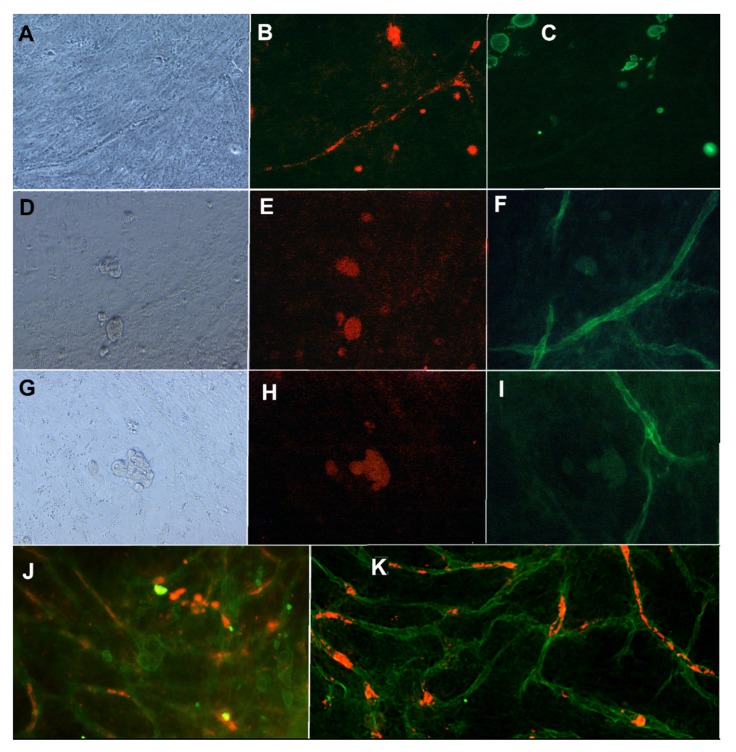
MCF7 cells grown in co-culture with vasculature. First row: (**A**) Phase contrast image, (**B**) anti-VWF (A568, red), and (**C**) anti-CK19 (FITC, green) staining. Second row: (**D**) Phase contrast image, (**E**) anti-CD44 (TRICH, red), and (**F**) anti-collagen IV (FITC, green) staining. Third row (**G**) Phase contrast image, (**H**) anti-ALDH1A1 (TRICH, red), and (**I**) anti-collagen IV (FITC, green). Images obtained with Nikon Eclipse Ti-s inverted fluorescence microscope with 20x objective. (**J**): MCF7 cells in co-culture with vasculature stained with anti-CK19 (FITC, green) and anti-VWF (A568, red). (**K**) same co-cultured stained with anti-collagen IV (FITC, green) and anti-ALDH1A1 (red). Imaged with Zeiss cell discoverer 7.

The results show that the mixture medium containing angiogenesis stimulating factors and the cancer cell-specific medium allows both the growth of vascular structures and the proliferation of cancer cells. Results showed that proper vasculature is maintained with 2.5 ng/mL of VEGF and 0.25 ng/mL of FGF-b in the co-culture with cancer cells. The intact basement membrane stained with anti-collagen IV (Figure 2F,I,K) and endothelial layer stained with anti-VWF (Figure 2B,J) were seen in the immunostainings of the co-culture. The intact endothelial cell tubule was located inside the collagen IV basement membrane (data not shown), as reported in earlier studies [11]. Positive staining for CD44, ALDH1A1, and CK19 were obtained also when MCF7 was grown on vascular structures (Figure 2). Figure 2 also shows the localization of CK19 positive MCF7 on and between VWF-stained tubules. The ALDH1A1 positive MCF7 cells are located near and on the collagen IV basement membrane.

**Figure 3:**
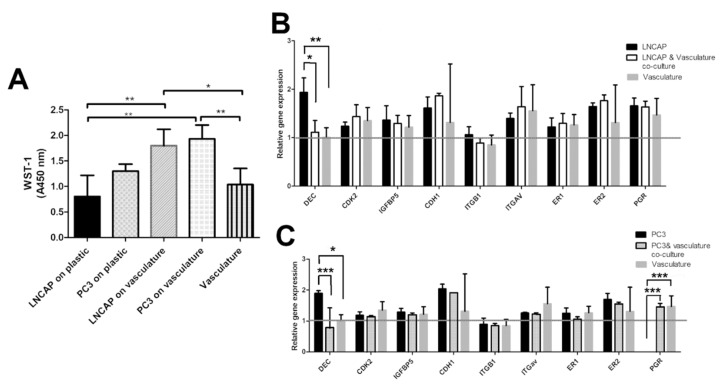
(**A**) Bar graph showing relative cell numbers in different cultures measured with WST-1. Difference between LNCAP and PC3 proliferation is smaller when grown on vasculature compared to plastic. Gene expression results of (**B**) LNCAP and (**C**) PC3 cells grown on plastic and on a vasculature platform. Grey line indicates the no change value (1) after normalization with housekeeping gene RPLP0. Results are obtained from 3 biological replicates (n = 3). Each sample contains total RNA from 6 parallel wells. * *p* < 0.05, ** *p* < 0.01 and *** *p* < 0.001.

The effect of vasculature as a platform for the proliferation of cancer cells was analyzed by WST-1 reagent. The result was utilized as a cell number of PC3 and LNCAP cells cultured on plastic and on vasculature. Based on the results, the vasculature seems to decrease the difference in the growth rate between LNCAP and PC3. In plastic, the LNCAP cells proliferated slower than PC3 (Figure 3). The medium for LNCAP on plastic is the same that is used for PC3 (commonly found in the literature), and the LNCAP and PC3 cultures on vasculature share the same medium. Due to the difference in media between plastic-grown and cells grown on vasculature, these cannot be compared to each other.

In addition to immunocytochemical staining, a video analysis of the cultures was obtained to provide more information on the interaction of the cell types. The cell movements and interaction between cells can be seen on the videos added as supplemental material (Video S1 MCF7 on plastic, Video S2 MCF7 on vasculature). Compared to the MCF7 grown on the plastic, the MCF7 grown on vasculature interacted more with other cells, moving more and extending more pseudopods, indicating that they receive more signals from the environment than on plastic.

In addition to morphological parameters, gene expression of cancer cell-related genes was studied. The selected genes present a selection of hormone receptors (estrogen receptor 1 and 2, progesterone receptor, PGR), fibrillogenesis genes (decorin, DEC), cell adhesion-related genes (integrin B1, integrin av, e-cadherin, CDH1), cell cycle-related genes (CDK2), and modulation of growth factor effects (IGFBP5). All of these genes are linked to cancer cells in different ways in the literature. Gene expression results on LNCAP show decreased expression of decorin (* *p* < 0.05) in the co-culture when compared to LNCAP grown on plastic. Other studied genes did not show a change in expression between co-culture and LNCAP monoculture. The gene expression results of PC3 show differences in expression of DEC and PGR between plastic grown PC3 and those grown in co-culture with vasculature. Decrease in DEC (*** *p* < 0.001) expression was seen in the PC3 co-culture compared to PC3 grown on plastic. Due to the lack of PGR expression in plastic-grown PC3 cells, the expression was significantly increased in the PC3 co-culture (*** *p* < 0.001) when compared to plastic-grown PC3 expression. Results were analyzed with two-way ANOVA followed by Bonferroni post-test.

### 2.3. Responses of Cancer Cells Grown on Plastic and Those Grown on Vascular Structures

To study the difference in responses of cancer cells grown on vasculature and with the traditional method on plastic, we exposed the cultures to five cancer drugs, doxorubicin, docetaxel, lapatinib, 5-fluorouracil and cyclophosphamide, at the concentration of 1 µM. The responses were quantified by WST-1 cell viability assay and by determining gene expression changes in the cultures. The WST-1 reagent measures the activity of mitochondria of cells. These results obtained from WST-1 analysis were interpreted here as relative living cell number or viability. As end-point analysis, this result may also indicate changes in proliferation. The gene expression changes were evaluated to see whether the cultures respond differently to drugs on a gene expression level. As a control, the vasculature alone without cancer cells was exposed to the tested drugs.

**Figure 4:**
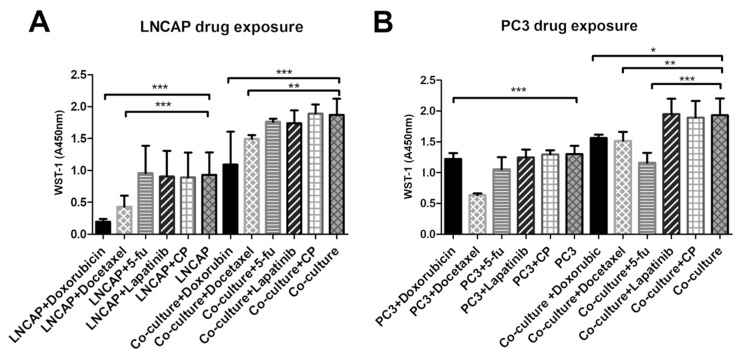
Viability of (**A**) LNCAP and (**B**) PC3 after exposure to five cancer drugs on plastic versus vasculature platform. Results are obtained from 3 biological replicates (n = 3). Each sample contained total RNA from 6 parallel wells. * *p* < 0.05, ** *p* < 0.01 and *** *p* < 0.001. 5-fu = 5-fluorouracil, CP = cyclophosphamide.

Overall, LNCAP had smaller cell numbers according to WST-1 assay on plastic than on vasculature. Both doxorubicin and docetaxel exposed cultures had significantly decreased viability (WST-1 result) than the unexposed control regardless of whether the LNCAP were grown on plastic or on vasculature (Figure 4).

From the tested chemicals, only docetaxel decreased the viability of the PC3 when cultured on plastic (Figure 4). However, when PC3 was grown on a vasculature platform, three of the tested chemicals (doxorubicin, docetaxel, and 5-fluorouracil) were found to significantly decrease viability compared to the unexposed culture (Figure 4). In the control (vasculature without cancer cells), no effect on viability was seen after the drug exposures when analyzed by WST-1 (data not shown).

Gene expression results showed some differences in responses between PC3 and LNCAP (Figures S1–S5). Doxorubicin exposed LNCAP cells had lower DEC, CDH1, and PGR expression on plastic compared to the unexposed LNCAP on plastic (*p* < 0.001 for all) and to doxorubicin exposed LNCAP grown on vasculature (*p* < 0.05, *p* < 0.01, and *p* < 0.01, respectively; Figure S1). Docetaxel, 5-fluorouracil, and lapatinib did not affect the expression of any of the studied genes in LNCAP (Figures S2–S4). Cyclophosphamide increased the expression of DEC in LNCAP grown on plastic compared to cyclophosphamide exposed LNCAP on vasculature (Figure S5).

Doxorubicin caused the upregulation of PGR in PC3 compared to unexposed PC3 on plastic (*p* < 0.001, Figure S1). Docetaxel upregulated DEC in PC3 on plastic compared to PC3 grown on vasculature (*p* < 0.05) and upregulated PGR in PC3 compared to unexposed PC3 on plastic (*p* < 0.001, Figure S2). 5-fluorouracil, Lapatinib, and cyclophosphamide increased PGR expression in PC3 grown on plastic compared to unexposed (*p* < 0.001). 5-fluorouracil and cyclophosphamide upregulated DEC expression in PC3 on plastic compared to PC3 on vasculature; *p* < 0.05 for 5-fluorouracil and cyclophosphamide and *p* < 0.01 for Lapatinib).

### 2.4. Angiogenic Induction Potential Assay for Cancer Cells

**Figure 5:**
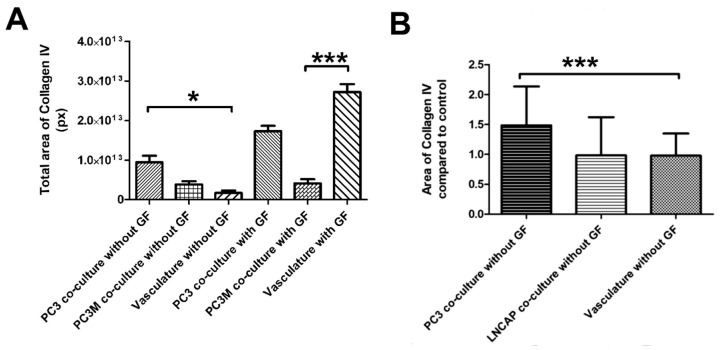
(**A**) Optimization of the angiogenesis induction test with PC3 and PC3M cells. Both show increased collagen IV induction compared to the vascular control (**B**) Optimized protocol without growth factors (GF) tested with PC3 and LNCAP cells. *** *p* < 0.05 and * *p* < 0.001.

We modified the protocol for vascularized cancer assay to enable the determination of the angiogenic induction potential of the cancer cells. First, we studied whether angiogenic growth factors VEGF and FGF-b are needed to aid the start of the angiogenesis process or not. With cell lines PC3 and PC3M, the induction of angiogenesis was quantifiable and higher than in control without angiogenic growth factors in the medium. (Figure 5A,B). The quantification of collagen IV-stained tubules showed PC3 to significantly induce angiogenesis compared to control (Figure 5A). Based on this result, there was no need to add growth factors to the medium for the angiogenic induction potential assay when working with cell lines. Cell line experiments were continued with this protocol lacking the external VEGF and FGF-b. When comparing the induction potential of LNCAP and PC3 with the optimized method, the results show that LNCAP does not increase the production of collagen IV-stained tubules, i.e., angiogenesis, and hence does not have high angiogenic potential (Figure 5B). The results of cell lines on the angiogenic induction potential test are found in Table 1.

To further test the functionality of this angiogenic induction test protocol and to provide a proof of concept for the test with primary cells, we isolated cells from eight patient cancer samples, solid and liquid, and tested their induction potential. At this point of assay development, the goal is merely to test whether or not cells can induce angiogenesis with this protocol. For providing this proof of concept for the assay, the number of patient samples is modest (eight) but adequate.

**Figure 6:**
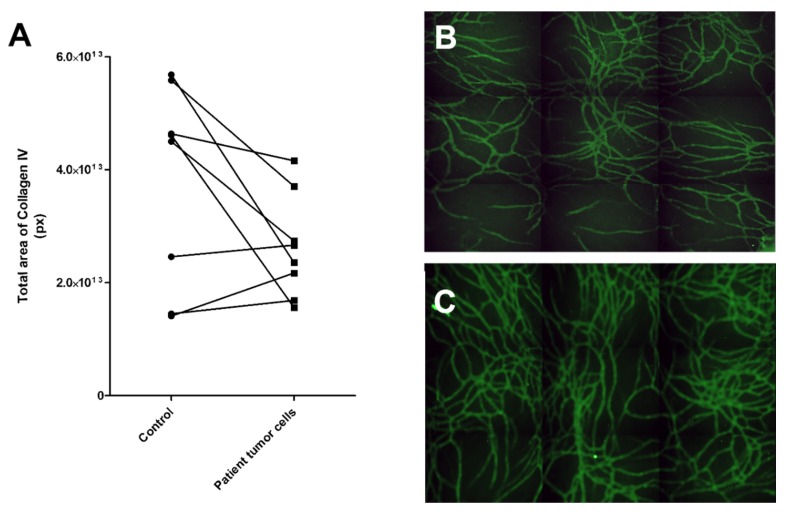
Testing of patient-derived cancer cells in the angiogenic potential assay. (**A**) Results of eight patient samples tested for their angiogenesis-induction potential. Three of the samples showed an increase in angiogenesis compared to the control, i.e., vasculature cells grown without patient sample cells. (**B**) Control vasculature without cancer cells had less vascular structures than (**C**) vasculature with patient sample 6. Control vasculature was cultured in the same medium as the experimental culture. The vasculature was stained with anti-collagen IV (FITC, green). Both B and C were obtained with automated imaging system (Cell-IQ) by imaging 3 x 3 grid with 10x objective and stitching these together to create a larger view of the well.

For the patient-derived cancer cells, we used 3:1 mixture of GCM or LCM (solid or liquid sample, see Section 4.3. for more information) and commercial EGM-2 medium (Lonza, contains VEGF and FGF-b) as the primary patient cells needed added growth factors to start the process of angiogenesis. Results showed that three of the eight samples induced more collagen IV stained tubule network formation than control, although two of these only modestly, compared to the control (Figure 6). The sample inducing angiogenesis most strongly was number 6, whereas patient samples 3 and 5 were mildly inductive. These patients were all in a progressive state of cancer (Table 2). Four of the samples were clearly not inducing angiogenesis. Sample number 4 induced only modestly less collagen IV tubule structures than the control. This was from ovarian cancer from solid tumor.

## 3. Discussion

In this study, our previously developed in vitro vasculature [11,14] was modified to (1) develop a test to identify those cancer cells which induce angiogenesis, and (2) function as a platform to culture cancer cells. For the first goal, we were successful in developing a protocol for estimating the angiogenesis induction potential of the cancer cells. For the second goal, the cancer cells grew well on the vascular platform and they stained positive for relevant markers. A proper network of vasculature was maintained in the culture with the cancer cells and expressed common blood vessel markers. The vascular component is used as a living scaffold for the cancer cells. In the drug tests, differences between responses of cells grown on plastic and vasculature were seen. Both culture set-ups were first optimized with known cancer cell lines and a pro-angiogenic potential test was also further tested on patient samples.

As the goal was to develop a culture method for personalized medicine and drug development, the developed method needed to be translatable into high throughput systems. For this, we first tested the scalability of the vasculature and succeeded in inducing and culturing the vasculature on 48- to 384-well plate formats. These vascular structures have previously been proven to contain lumen surrounded by endothelial layer, basement membrane, pericytes, and junction proteins [11]. The use of hASC in the co-culture greatly contributes to this in vivo likeness of the vascular structures. Stem cells in the population can differentiate into pericyte-like cells and the population produces relevant ECM components for the vasculature to grow proteins [11]. In addition to being scalable to 384-well plates, the methods presented here have a relatively short culture time, 7 days. This allows the real possibility to utilize these protocols in selecting the right drugs for the patient.

Cancer cells utilized here showed positivity for CD44, ALDH1A1, and CK19 markers when grown on vasculature platform. CK19 is an epithelial-specific marker and is normally expressed in the gastroenteropancreatic and hepatobiliary tracts and can be found in tumors related to these tissues [23]. CK19 expression is also detected in MCF7 [15] and it was used to see how the MCF7 localized compared to vasculature. In Figure 2, it is clearly seen that the MCF7 cancer cell clusters stained with CK19 are growing on top of and in between the vascular structures and thus can interact with the vasculature. This allows natural cell–cell interactions and the exchange of substances.

The CD44 antigen is a cell-surface glycoprotein involved in cell–cell interactions, cell adhesion, and migration [16]. CD44 has been reported as a cell surface marker for some breast and prostate cancer stem cells [24]. Tumor endothelial cells with high aldehyde dehydrogenase activity have been shown to have a highly angiogenic phenotype and to be drug-resistant [25]. In Figure 2, there is positive staining with ALDH1A1, which co-locates with the collagen IV staining. This might indicate that the MCF7 has attached to the vascular structures or has integrated into the vascular structures. However, further studies on this phenomenon would be needed. Li et al. reported that MCF7 expressed ALDH1 but not CD44 in their experiments when grown on plastic in a similar medium as used here for MCF7 cells [26]. In our results, both CD44- and ALDH1-positive MCF7 cells were found. Based on the supplemental videos, the presence of cell–cell interaction marker CD44 in MCF7 is not surprising as the interaction between vasculature cells and MCF7 is clear, as discussed below.

The interaction and increased movement of cancer cells on vasculature are also seen in the supplemental videos (Videos S1 and S2). This kind of interaction allows activation of more in vivo-like characteristics as the interaction between tumor cells and vasculature in vivo has been found important on many levels, including migration, proliferation, and sprouting [27]. The communication between cells happens in many ways. The interaction between cancer cells and endothelial cells is at least partly mediated by the vesicles secreted by endothelial cells [28]. Tumor-derived extracellular vesicles internalized by endothelial cells have been shown to be one mechanism for tumor cells to induce angiogenesis by enhancing EC migration, proliferation, and sprouting [27]. These tumor exosomes have been reported to transfer miRNAs when taken up by ECs, which contributes to the induction of angiogenesis [29,30].

When we studied the proliferation of the cancer cell, we detected that the difference in proliferation rates between LNCAP and PC3 were smaller on vasculature compared to cells grown on plastic. On plastic, the growth rates were clearly different, LNCAP having a slower proliferation rate. Due to the difference in media between plastic-grown and cells grown on vasculature, these platforms cannot be compared to each other. However, it has been shown previously that many genes that promote rapid growth and proliferation are often upregulated in cell lines in the 2D culture environment and the expression of genes that limit growth and proliferation are repressed in 2D-adapted cell lines compared to their corresponding tissue origins [6]. This supports our hypothesis that by adding the vasculature, the high throughput 2D culture can be brought closer to in vivo and hence make it more relevant and reliable.

The differences between LNCAP and PC3 were clear on the phase contrast examination of the cultures containing vasculature. LNCAP cells created clear masses, whereas the more aggressive PC3 cells blended in the cell population of the co-culture. On plastic, both cells grew in monolayer, in some cases creating very large masses.

The gene expression results show that decorin expression in LNCAP and PC3 was decreased when grown on vasculature. Decorin is a small leucine-rich proteoglycan expressed in connective tissue that modulates collagen fibrillogenesis in angiogenesis [31,32]. In addition, decorin is known to suppress prostate tumor growth through inhibition of epidermal growth factor and androgen receptor pathways [33]. Earlier in vitro studies showed that reduced decorin expression facilitates tumorigeneses and growth of breast [34], ovarian [35], and pancreatic cancers [36], among others [37].

Progesterone receptor was not identified in the plastic grown PC3 cells but was expressed in co-culture and vasculature alone. Hence the increase in the progesterone receptor was significantly upregulated in co-culture compared to PC3 grown on plastic with traditional methods, including the commonly-used medium. Because the progesterone receptor expression is high in vasculature control, it seems that the progesterone receptor expression is coming from the vasculature cells, i.e., hASC and HUVEC. Peripheral veins have been shown to contain progesterone receptors in patient samples [38], which further confirms the in vivo likeness of the platform for cancer cells. Progesterone receptor positive stromal cells have been shown to inhibit prostate cancer cell migration and invasion [39]. However, in our results, this kind of reduction in cell functions or movement was not seen in our results (Videos S1 and S2).

Drug development is expensive, and hence relevant and reliable in vitro models are needed for preclinical studies. There is also a need for predictive functional tests for personalized selection of cancer drugs for patients. Exposing cultured patient-derived cancer cells with cancer drugs is being used more and more frequently. Better predicting cell culture models are also important there. Low rates of in vivo translation of new therapeutic agents are partially due to the use of too-simplistic 2D monolayer culture systems for in vitro drug discovery and development [5,40]. Contrary to our results, 2D cultures are often more sensitive to drug therapies, yielding many false-positive results in 2D drug screens [5,40]. In our results, the LNCAP cells responded in a similar way on vasculature and on plastic to the selected drugs, and PC3 cells were sensitive to multiple drugs (docetaxel, doxorubicin, 5-fluorouracil) on vasculature compared to one drug (Docetaxel) when grown on plastic. Docetaxel is an antineoplastic agent that acts by disrupting the microtubular network in cells that is essential for mitotic and interphase cellular functions. Hence, the activity of cells on vasculature (mobility, pseudopod formation, cell–cell interactions) could lead to the effect of docetaxel being seen earlier in these cells. Doxorubicin interacts with DNA by intercalation and inhibition of macromolecular biosynthesis [41]. 5-fluorouracil causes deficiency in dTMP, hence rapidly dividing cancerous cells undergo cell death due to inhibition of thymidylate synthase and hence lack on thymine [42]. The positive result with fluorouracil is hence explained by the higher activity (mobility and interaction with other cells seen on the supplemental videos) of cells grown on vasculature.

The angiogenic induction potential test was successfully developed. We were able to quantify the deposition of collagen IV and used it as a marker for angiogenesis. The amount of collagen IV correlated well with the known angiogenic features of LNCAP and PC3. Androgen-deprived LNCAP cells have been shown to have diminished VEGF production [43] and were found not to produce FGF-b in contrast PC3, which produced FGF-b [44]. The VEGF production has been shown to be higher in PC3 than in LNCAP [21]. The result also correlates with the metastatic potential of these cell lines, and the PC3 are known to be highly metastatic and LNCAP non-metastatic [45]. Other studies have also shown the connection between metastatic potential and VEGF production of human prostate cancer cells [46]. Thus, VEGF seems to play an important role in prostate cancer metastasis. On the other hand, also the amount of collagen IV has been shown to correlate with tumor burden. This was shown by selectively targeting collagen IV in the cancer cell microenvironment, which was found to reduce tumor burden [47].

One major finding in this study is that the differences in angiogenesis induction were seen also in patient-derived cancer cells. Although we could not see the induction of angiogenesis in ovarian cancer samples, which are in general treated with angiogenesis inhibitors, the sigma adenocarcinoma sample induced angiogenesis and is also commonly treated with angiogenesis inhibitors. At this time, we have no method for verifying in practice that three of the tested patients would benefit from the anti-angiogenic treatment; the difference between these three samples was clear compared to the other five samples. Also, all three samples were from patients with a confirmed progressive stage of the disease. This exciting result shows that this angiogenic induction potential assay could be used as a screening method for personalized treatment planning.

The responses of the cancer cells grown on the vasculature platform need further studying. Here only selected cancer cell lines and genes were studied, hence a broader study would be merited next to confirm that the responses and expression changes are corresponding to those in vivo. The gene expression results and morphological observations confirm the interaction of the cancer cells and that the vasculature indeed has an effect on the cancer cells. This cancer assay will be further studied and optimized, especially for high throughput drug efficacy studies. The angiogenic potential test will be validated next to ensure the correct responses and to allow the use of this in vitro test more broadly.

One limitation of the study is the lack of flow and blood substituent in the vascular structures. With the addition of this component to the culture, the metastatic potential of the cancer cells could be studied on the platform. The use of microvascular endothelial cells could also be considered in the future as they would be more relevant cell type for these capillary size vascular structures. HUVEC in these presented protocols could be replaced with microvascular endothelial cells. With the use of tissue-specific microvascular endothelial cells, different cancer environments could be developed and the interaction of these endothelial cells and cancer cells could be studied on the specific tissue level. Limitations of this study also include the low number of patient samples. However, at this point of protocol development, the goal is to provide a proof-of-concept for the angiogenic induction potential test, which was achieved with the current set of samples. In the future, more extensive patient studies are needed, which could include drug trials. A more extensive gene expression study would also be merited to find out the pathways that are activated in the interaction with vasculature. The interaction taking place between cancer cells and vasculature should also be studied in more detail. These protocols provide interesting options for new lines of studies, especially with human primary cells.

## 4. Materials and Methods

### 4.1. Ethical Considerations

This in vitro study conforms to the ethical principles outlined in the Declaration of Helsinki. The human adipose tissue samples were excess material of surgical operations, human umbilical cords were from casarean sections and tumor samples from removal surgeries, all received with written informed consents at Tampere University Hospital, Tampere, Finland. The use of tumor samples, hASC, and HUVEC was approved by The Regional Ethics Committee of Tampere University Hospital’s Responsibility Area with permit numbers of R16127, R15161, and R15033, respectively.

### 4.2. The Isolation, Culture and Quality Control of Human Primary Cells hASC and HUVEC

The isolation, culture, and quality control of hASC and HUVEC were performed as described previously [11,48]. hASC were cultured in DMEM/F12 supplemented with 10% human serum (HS, Lonza Group Ltd., Basel, Switzerland) and 1% L-glutamine (Gibco, Carlsbad, CA, USA). hASC used in the study were heterogenous cell populations obtained by isolating the stromal vascular fraction cells of human adipose tissue. HUVEC were expanded in a Endothelial Cell Growth Medium-2 Bullet Kit (EGM-2, Lonza). In the co-culture, hASC was at passage 2, and HUVEC at passage 4. Both cell types were free of mycoplasma, as tested with MycoAlert^®^ Mycoplasma Detection Kit (Lonza). hASC were characterized for markers CD73, CD90, and CD105 (BD biosciences, Franklin Lakes, NJ, USA) with flow cytometer FACSCAnto II (BD biosciences), as previously published [11], before experimental use. In the co-culture, hASC was at passage 2, and HUVEC at passage 4.

### 4.3. Isolation of Primary Cancer Cells

Liquid patient samples (pleural effusion or ascites fluid) were treated as follows. Liquid was centrifuged at 200× *g* for 10 min, supernatant was removed, and pellets were combined with aid of PBS (Gibco). Pellets were suspended into PBS and run through a strainer of 100 µm. This was then centrifuged at 200× *g* for 5 min, supernatant was removed, and pellets were suspended to liquid cancer medium (LCM, Table 3), and they were expanded in 75 cm^2^ culture bottles until used for co-culture.

Solid tumor samples were treated as follows. A human tumor dissociation kit (Miltenyi biotec, Bergisch Gladbach, Germany) was utilized in isolating the primary tumor cells. The tumor sample was manually dissected into 2–4 mm pieces. Pieces were placed into a gentleMACS C tube (Miltenyi Biotec) with enzyme mix provided in the kit. A tube was placed on a gentleMACS dissociator with heaters (Miltenyi Biotec). The samples were run with the 37C_h_TDK_2 program. After the run, a short centrifugation was performed to spin down the sample. The sample was resuspended and strained through a 70-µm MACS smartstrainer (Miltenyi Biotec) with the aid of RPMI1640 (Gibco). Suspension was centrifuged at 250× *g* for 7 min. Supernatant was aspirated, cells were resuspended into the general cancer cell medium (GCM, Table 3) and they were expanded in 75 cm^2^ culture bottles until used for co-culture.

### 4.4. Expansion of Cancer Cell Lines

LNCAP, PC3, MCF7, and PC3M cell lines were utilized (Table 1). Media used for culturing the cell lines are found in Table 3.

### 4.5. Establishment of the Co-Culture of Cancer Cells and Vascular Network for Drug Exposure

Co-culture of hASC and HUVEC was established as published previously [11,14]. The co-culture system was scaled for 48-, 96-, and 384-well plate formats to allow more variety and high throughput studies. The seeding cell numbers which were found optimal are listed in Table 4. Day 0, hASC were seeded in EGM-2 (Lonza), followed by seeding of HUVEC in EGM-2 (Lonza). The cancer cells were seeded on top of hASC and HUVEC on the same day in their own cancer cell line-specific medium described above. The ratio of EGM-2 (Lonza) to cancer medium at the end of the day was 4:1. On day 1, a 1:1 mix of angiogenesis stimulating medium (ASM, Table 3) and cancer cell specific medium was applied to the co-culture. On day 4, the medium was replenished. The co-culture was grown for 7 days in total. Each experiment included vascular control (co-culture without cancer cells). The cancer cells grown on plastic were seeded at the same density as for the co-cultures, but the medium used was the cancer cell-specific medium (Table 3).

Drug treatments were added to the co-cultures on day 1. LNCAP and PC3 cell lines were utilized. Doxorubicin hydrochloride (44583, Sigma, Saint Louis, MO, USA), Docetaxel (01885, Sigma), cyclophosphamide monohydrate (93813, Sigma), lapatinib (CDS022971, Sigma) and 5-fluorouracil (03738, Sigma), all at concentrations of 1 µM, were utilized. The untreated controls were exposed to 0.5% DMSO as this was also the fixed concentration of DMSO in the drug treatment co-culture wells. In drug tests, each experiment included drug-treated and untreated controls of the co-culture and drug-treated vascular control to determine the toxicity to vasculature. At day 4, the medium and drug dilutions were replenished.

At day 7, analyses were performed, including gene expression analysis by qPCR, WST-1 analysis, and immunostaining. WST-1 (Roche Diagnostics, Basel, Switzerland) was incubated for 1.5 h. Absorbance was measured at 450 nm with a Varioskan flash multimode reader (Thermo Fischer Scientific, Waltham, MA, USA). The WST-1 results are used to depict relative cell numbers and viability. After WST-1 analysis, immunostaining was performed as described below.

### 4.6. Modifying the Co-Culture System for Testing the Angiogenic Potential of Cancer Cells

The protocol for co-culturing cancer cells and the vascular network was modified to determine the effect of cancer cells on the vasculature. Only modification to the above protocol was the medium used from day 1 forward. On day 1, either cancer cell-specific medium or 3:1 mix of general cancer medium (GCM, Table 3) and ASM (Table 3) was applied to the co-culture. On day 4, the medium was changed to a fresh one. The co-culture was cultured for 7 days in total. The vasculature control culture (without cancer cells) was treated with the same protocol as the experimental culture. Experiments were performed on a 96-well plate. The functionality of the protocol was tested by utilizing pro-angiogenic PC3 and PC3M cell lines and non-inducer cell line LNCAP (Table 1).

### 4.7. Testing the Angiogenic Potential of Patient Derived Cancer Cells

The test medium was 3:1 mix of GCM or LCM depending on the type of the sample (liquid or solid sample, Table 3) and EGM-2 (Lonza). Cancer cells were derived from 8 patient samples. These were tested in the Angiogenic Induction Potential Test. The characteristics of these patients are collected in Table 2. The control vasculature was exposed to the same medium that was utilized in the cancer cell-containing samples.

### 4.8. Immunofluorescence Staining

To visualize the vascular structures and cancer cells, immunocytochemical staining was performed. Co-cultures were fixed with 70% ethanol at day 7. After fixation, the cells were permeabilized with 0.5% Triton-X100 (MP Biochemicals, Solon, OH, USA) and non-specific binding sites were blocked with 10% BSA (Roche Diagnostics). Primary and secondary antibodies were applied in 1% BSA (Roche). Primary antibodies utilized were rabbit anti-human VWF IgG (1:100, F3520, Sigma and A0082, DAKO), basement membrane marker mouse anti-human collagen IV (anti-COLIV, clone COL-94, 1:500, C1926, Sigma), rabbit anti-human ALDH1A1 (1:250, ab52492, Abcam, Cambridge, UK), rabbit anti-human CD44 (1 µg/mL, ab157107, Abcam), mouse anti-human CK19 monoclonal antibody (2 µg/mL, RCK108, Thermo fisher scientific), secondary antibodies anti-rabbit IgG A568 (1:400, ab175471, Abcam), and anti-mouse IgG fluorescein isothiocyanate (FITC, 1:100, F4143, Sigma). A drop of Fluoroshield™ with DAPI histology mounting medium (Sigma) was added into the emptied wells to stain the nuclei and mount the cultures.

### 4.9. Microscopy and Quantification of Vascular Structures

Microscopic imaging was done with a Nikon Eclipse Ti-s inverted fluorescence microscope (Nikon, Tokyo, Japan) and a Nikon digital sight DS-U2 camera (Nikon) and images were further processed with NIS Elements (Nikon), ZEN 2012 software (Carl Zeiss, Oberkochen, Germany), and Adobe Photoshop CS3 software (Adobe Systems Incorporated, San Jose, CA, USA).

In order to investigate the area of tubular networks, Fcell cultures were imaged with Cell-IQ (CM Technologies Oy, Tampere, Finland), as described earlier (Huttala et al. (2018)). Briefly, culture plates were imaged with 10× objective and 3 × 3 grid per well. Grids were stitched together with Cell-IQ Analyzer (CM Technologies Oy) and further analyzed with ImageJ software (The National Institutes of Health (NIH), Bethesda, MD, USA). Images were converted to an 8-bit grayscale, background was substracted, and binary threshold was adjusted to determine the total tubule area in pixels.

### 4.10. PCR Analysis

Expressions of cancer-related genes (decorin, IGFBP5, Integrin B1, Integrin av, estrogen receptor 1, estrogen receptor 2, e-cadherin, progesterone receptor, CDK2) were studied as follows. Each RNA sample was obtained by collecting the cell lysis from 6 parallel wells. The experiments were repeated three times (3 biological replicates, n = 3), one sample per repeat. RNA isolation was performed using the Purelink RNA mini kit (Invitrogen, Carlsbad, CA, USA) and PCR was performed with ITaq universal SYBR green one-step kit (Biorad, Hercules, CA, USA), both according to the manufacturers’ instructions. Each reaction contained 30 ng of the template, and the primer concentration was 300 nM. Primer sequences and annealing temperatures can be seen in Table 5. Run contained 10-min incubation at 50 °C, 1-min incubation at 95 °C, and 45 cycles of 10 s at 95 °C, and 30 s at 60 °C. This was followed by melt curve analysis from 65 °C to 95 °C. PCR amplification was performed with a CFX96 real-time system (Biorad). RPLP0 was used as the housekeeping gene. Each PCR cycle contained one well per sample.

### 4.11. Statistical Analyzes

All calculations were made with Microsoft Excel 2010 (Microsoft Corporation, Redmond, WA, USA) and statistical analyses performed in GraphPad Prism (GraphPad Software Inc., La Jolla, CA, USA). Results are depicted as mean ± standard deviation. Differences were considered significant when * *p* < 0.05 (significant), ** *p* < 0.01(very significant) and *** *p* < 0.001 (extremely significant). Results from cell line induction optimization (n = 10) and PC3 and LNCAP induction results (n = 30) were subjected to Kruskal–Wallis with Dunns’ post-test. The gene expression results (n = 3) were subjected to two-way ANOVA followed by Bonferroni’s post-test. WST-1 results on comparing plastic and on vasculature as culture platform had n = 6 and no statistical analysis was performed. WST-1 results in drug exposure experiments (n = 6), were subjected to one-way ANOVA, followed by Sidak’s multiple comparison test.

## 5. Conclusions

We have developed two new protocols for culturing cancer cells on vascular structures composed of human derived cells. We demonstrate differences in drug responses between cancer cells cultured with traditional medium on plastic and those grown on in vitro vasculature. In addition, we demonstrate that the protocol presented here can be used to detect cancers that induce angiogenesis. Hence, these are interesting new culture methods and could be beneficial for drug development purposes, cancer research, and personalized selection of drugs for cancer patients. The protocols and their defined components can be easily modified. This facilitates, for example, addition of immune cells into the co-culture. The vascular platform for culturing cancer cells is adaptable to a 384-well format for high throughput screening. The co-culture of vasculature and cancer cells will be further characterized and tested with a larger set of patient samples in the future.

## Figures and Tables

**Table 1:** Information about the cell lines used in the study and results obtained from the angiogenic induction potential test.

Cell Line	Origin	Other Info	Reference	Angiogenesis Induction in Our Assay
MCF7	Invasive breast ductal carcinoma: pleural effusion	Low tumorigenicity, poor angiogenic potential	[17,18]	-
LNCAP	human prostate adenocarcinoma: lymph node metastasis	Noninvasive	[19]	No induction
PC3	bone metastasis of grade IV of prostate cancer	High tumorigenicity, Secreting VEGF	[20,21]	Induction
PC3M	Highly metastatic cell line derived from PC3	Highly tumorigenic, angiogenic but not secreting VEGF165	[22]	Induction

**Table 2:** Characteristics of the samples received from patients and results obtained from the angiogenic-induction potential test. Those samples which differ only mildly from control are marked in parenthesis.

Sample Number	Form of Sample	Cancer Type/Origin	Drugs Received by the Patient	Angiogenesis Inducer
1	Ascites	Progressive, originating from colon, adenocarcinoma	Regorafenib	No
2	Ascites	CA12s increasing ascites increasing, ovarian cancer	Bevacizumab, Paclitaxel, Carboplatin, Letrozol	No
3	Pleural effusion	Metastatic thyroid cancer, progressive	Sorafenib	(Yes)
4	Solid tumor	Ovarian cancer		(No)
5	Pleural effusion	Breast cancer, progressive	Eribulin	(Yes)
6	Ascites	Sigma adenocarcinoma, progressive	Capecitabine, Oxaliplatin	Yes
7	Solid tumor	Ovarian cancer		No
8	Pleural effusion	Unknown		No

**Table 3:** Media used in the study.

**Angiogenesis stimulating medium (ASM)**	DMEM/F12 2.56 mM L-glutamine 0.1 nM 3,3′,5-Triiodo-L-thyronine sodium salt ITS^TM^ Premix: 6.65 µg/mL insulin 6.65 µg/mL Transferrin 6.65 ng/mL selenious acid 1% Bovine serum albumin (BSA) 2.8 mM Sodium puryvate 200 µg/mL Ascorbic acid 0.5 µg/mL Heparin 2 µg/mL Hydrocortisone/ cortisol 10 ng/mL Vascular endothelial growth factor (VEGF) 1 ng/mL Fibroblast growth factor (FGF-b)	Gibco Gibco Sigma BD Roche Gibco Sigma Sigma Sigma R&D systems (Minneapolis, MN, USA) R&D Systems
**General cancer cell medium (GCM)**	DMEM/F12 2.56 mM L-glutamine 5% human serum	Gibco Gibco Lonza
**Liquid cancer sample medium (LCM)**	DMEM/F12 2.56 mM L-glutamine 10% Supernatant from the isolation of the cells	Gibco Gibco
**MCF7 medium**	DMEM/F12 supplemented 10% fetal bovine serum 1% L-glutamine 10 ng/mL insulin	Gibco Gibco Gibco Sigma
**PC3, LNCAP, and PC3M medium**	RPMI1640 (containing 1mM L-glutamine) 1% penicillin-streptomycin (pen/strep) 10% fetal bovine serum	Gibco Gibco Gibco

**Table 4:** Number of seeded cells for different well plate formats.

Well Plate	Growth Area	Cancer cells Per Well	hASC Per Well (20,000 Cells/ cm^2^)	HUVEC Per Well (4100 Cells/ cm^2^)
48	1.1 cm^2^	10,000	22,000	4500
96	0.33 cm^2^	5000	6600	1350
384	0.13 cm^2^	1000	2600	533

**Table 5:** Primer sequences used in the one-step RT–qPCR.

Gene	Abbreviation	Primer Sequence
**Decorin**	DEC	GGACCGTTTCAACAGAGAGG Reverse GAGTTGTGTCAGG GGGAAGA
**IGFBP5**		GCTCAGGCTTGAGGGTTTCT Reverse primer AGAGCGAGAGTGCAGGGATA
**Integrin B1**	ITGB1	GCCGCGCGGAAAAGATGAA Reverse primer TGCTGTTCCTTTGCTACGGT
**Integrin av**	ITGAV	CGGAGGGAAGCAAAGGACC Reverse primer GTAGCAGGAGTCCCGAGAGA
**Estrogen receptor 1**	ER1	CGTCGCCTCTAACCTCGG Reverse primer AGCTCGTTCCCTTGGATCTG
**Estrogen receptor 2**	ER2	GGCTGCGAGAAATAACTGCC Reverse AATGCGGACACGTGCTTTTC
**E-cadherin**	CDH1	CAGTTCAGACTCCAGCCCG Reverse CACCGTGAACGTGTAGCTCT
**Progesterone receptor**	PGR	ACCCGCCCTATCTCAACTACC Reverse AGGACACCATAATGACAGCCT),
**CDK2**		GACACGCTGCTGGATGTCA Reverse CAGAAAGCTAGGCCCTGGAG
**RPLP0**		AATCTCCAGGGGCACCATT Reverse CGCTGGCTCCCACTTTGT

## Supplementary Materials

Supplementary materials can be found at https://www.mdpi.com/1422-0067/21/5/1833/s1. Figure S1. Full gene expression results. LNCAP and PC3 cells exposed to Doxorubicin. Red line indicates no change compared to housekeeping RPLP0. * *p* < 0.05 (significant), ** *p* < 0.01(very significant) and *** *p* < 0.001 (extremely significant). Figure S2. Full gene expression results. LNCAP and PC3 cells exposed to Docetaxel. Red line indicates no change compared to housekeeping RPLP0. * *p* < 0.05 (significant) and *** *p* < 0.001 (extremely significant). Figure S3. Full gene expression results. LNCAP and PC3 cells exposed to 5-fluorouracil. Red line indicates no change compared to housekeeping RPLP0. * *p* < 0.05 (significant) and *** *p* < 0.001 (extremely significant). Figure S4. Full gene expression results. LNCAP and PC3 cells exposed to Lapatinib. Red line indicates no change compared to housekeeping RPLP0. ** *p* < 0.01(very significant) and *** *p* < 0.001 (extremely significant). Figure S5. Full gene expression results. LNCAP and PC3 cells exposed to Cyclophosphamide (CP). Red line indicates no change compared to housekeeping RPLP0. * *p* < 0.05 (significant) and *** *p* < 0.001 (extremely significant). Video S1. Video from the culture of MCF7 grown on plastic. Only small movement and no significant pseudopod formation is observed. Video filmed with Cell-IQ. Video S2. Video from the culture of MCF7 grown on in vitro vasculature. Cells are more active than those on plastic. Cell-cell interaction, movement and pseudopod formations are observed in the culture. Video filmed with Cell-IQ.

## Abbreviations

ASM	Angiogenesis stimulating medium
GCM	General cancer medium
LCM	Liquid cancer sample medium
BSA	Bovine serum albumin
VEGF	Vascular endothelial growth factor
FGF-b	Fibroblast growth factor
hASC	human adipose stromal cell
HUVEC	human umbilical vein endothelial cell
DEC	Decorin
ITGB1	Integrin B1
ITGAV	Integrin av
ER1	Estrogen receptor 1
ER2	Estrogen receptor 2
CDH1	E-cadherin
PGR	Progesterone receptor
5-fu	5-fluorouracil
CP	Cyclophosphamide
DX	Doxorubicin
DC	Docetaxel
LA	Lapatinib
CK19	Cytokeratin 19
EGM-2	Endothelial Cell Growth Medium-2 BulletKit
VWF	Von Willebrand factor
