# Supplementary material for: In Vitro Vascular Network Modified to Function as Culture Platform and Angiogenic Induction Potential Test for Cancer Cells
Source: Int J Mol Sci. 2020 Mar 6;21(5):1833. doi: 10.3390/ijms21051833 (PMC7084873; doi:10.3390/ijms21051833)
Supplement: Supplementary file 1 [file ijms-21-01833-s001.zip › ijms-723674-supplementary/Revised Supplementary files_Huttala/Supplementary file captions R.docx]

Figure S1. Full gene expression results. LNCAP and PC3 cells exposed to Doxorubicin. Red line indicates no change compared to housekeeping RPLP0. *p<0.05 (significant), **p<0.01(very significant) and ***p<0.001 (extremely significant).

Figure S2. Full gene expression results. LNCAP and PC3 cells exposed to Docetaxel. Red line indicates no change compared to housekeeping RPLP0. *p<0.05 (significant) and ***p<0.001 (extremely significant).

Figure S3. Full gene expression results. LNCAP and PC3 cells exposed to 5-fluorouracil. Red line indicates no change compared to housekeeping RPLP0. *p<0.05 (significant) and ***p<0.001 (extremely significant).

Figure S4. Full gene expression results. LNCAP and PC3 cells exposed to Lapatinib. Red line indicates no change compared to housekeeping RPLP0. **p<0.01(very significant) and ***p<0.001 (extremely significant).

Figure S5. Full gene expression results. LNCAP and PC3 cells exposed to Cyclophosphamide (CP). Red line indicates no change compared to housekeeping RPLP0. *p<0.05 (significant) and ***p<0.001 (extremely significant).

Video S1. Video from the culture of MCF7 grown on plastic. Only small movement and no significant pseudopod formation is observed. Video filmed with Cell-IQ.

Video S2. Video from the culture of MCF7 grown on in vitro vasculature. Cells are more active than those on plastic. Cell-cell interaction, movement and pseudopod formations are observed in the culture. Video filmed with Cell-IQ.
