# Supplementary figures and images for: In Vitro Vascular Network Modified to Function as Culture Platform and Angiogenic Induction Potential Test for Cancer Cells
Source: Int J Mol Sci. 2020 Mar 6;21(5):1833. doi: 10.3390/ijms21051833 (PMC7084873; doi:10.3390/ijms21051833)

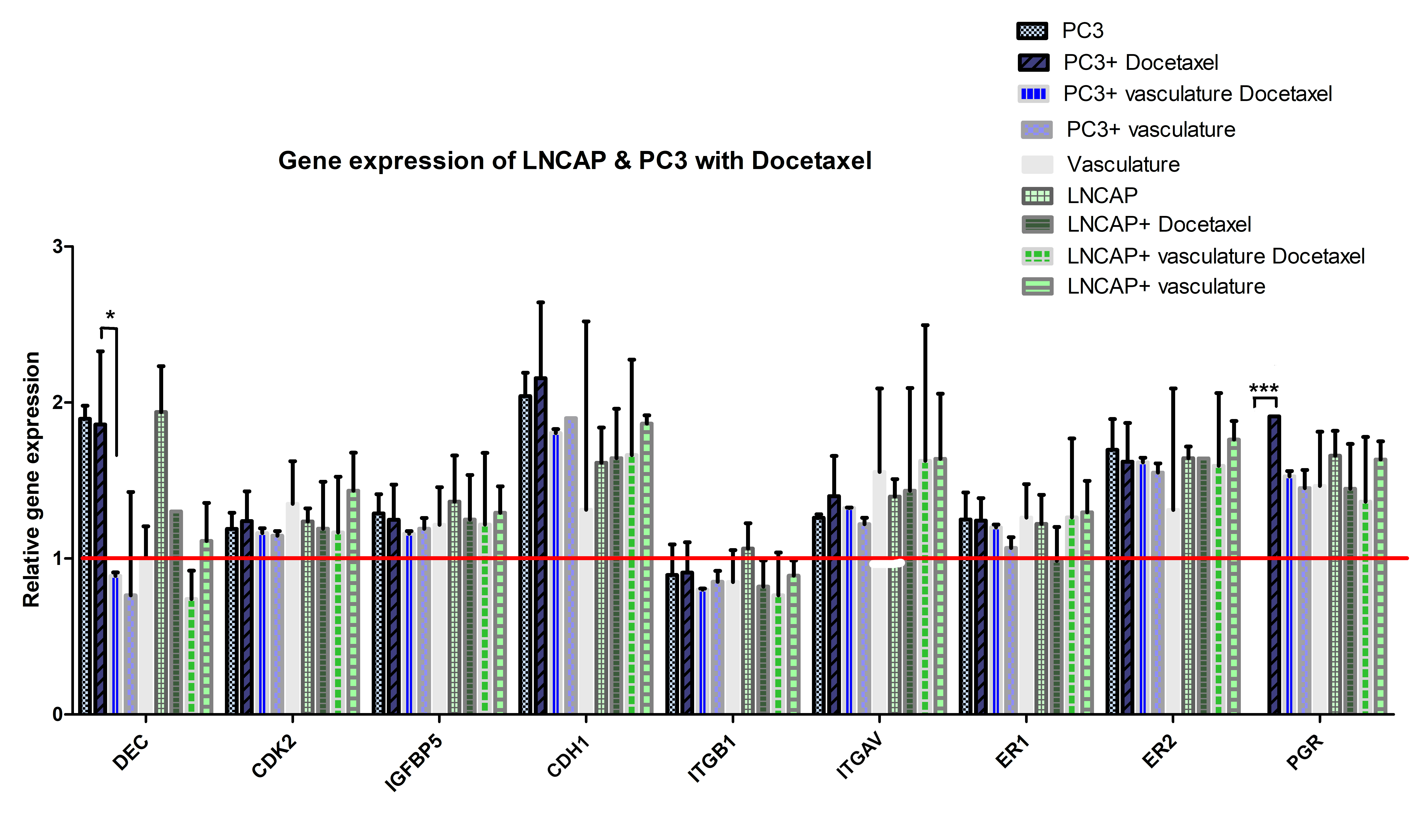

Supplement: Supplementary file 1 [file ijms-21-01833-s001.zip › ijms-723674-supplementary/Revised Supplementary files_Huttala/Figure S2_Docetaxel R.tif]

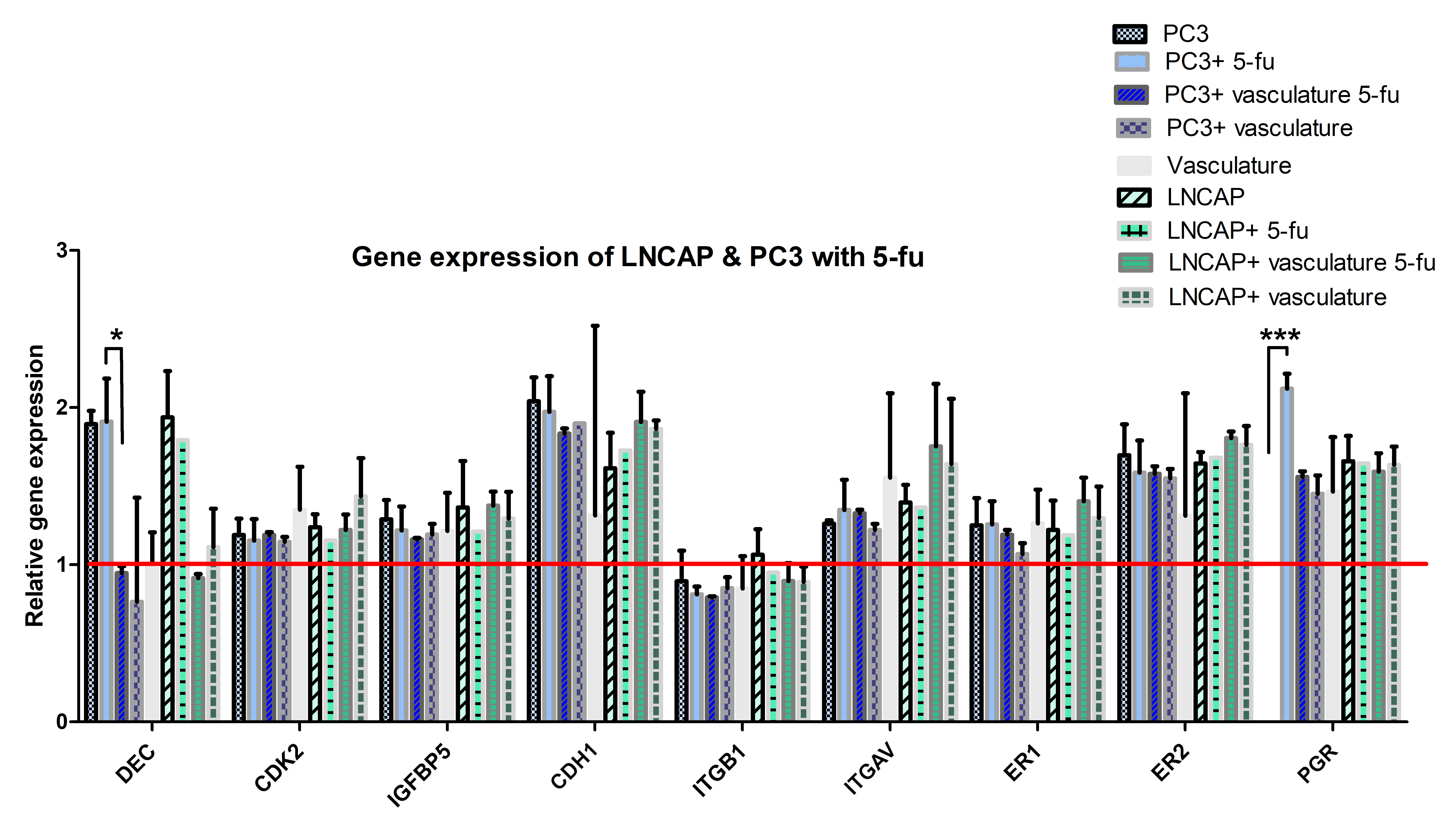

Supplement: Supplementary file 1 [file ijms-21-01833-s001.zip › ijms-723674-supplementary/Revised Supplementary files_Huttala/Figure S3_5fu R.tif]

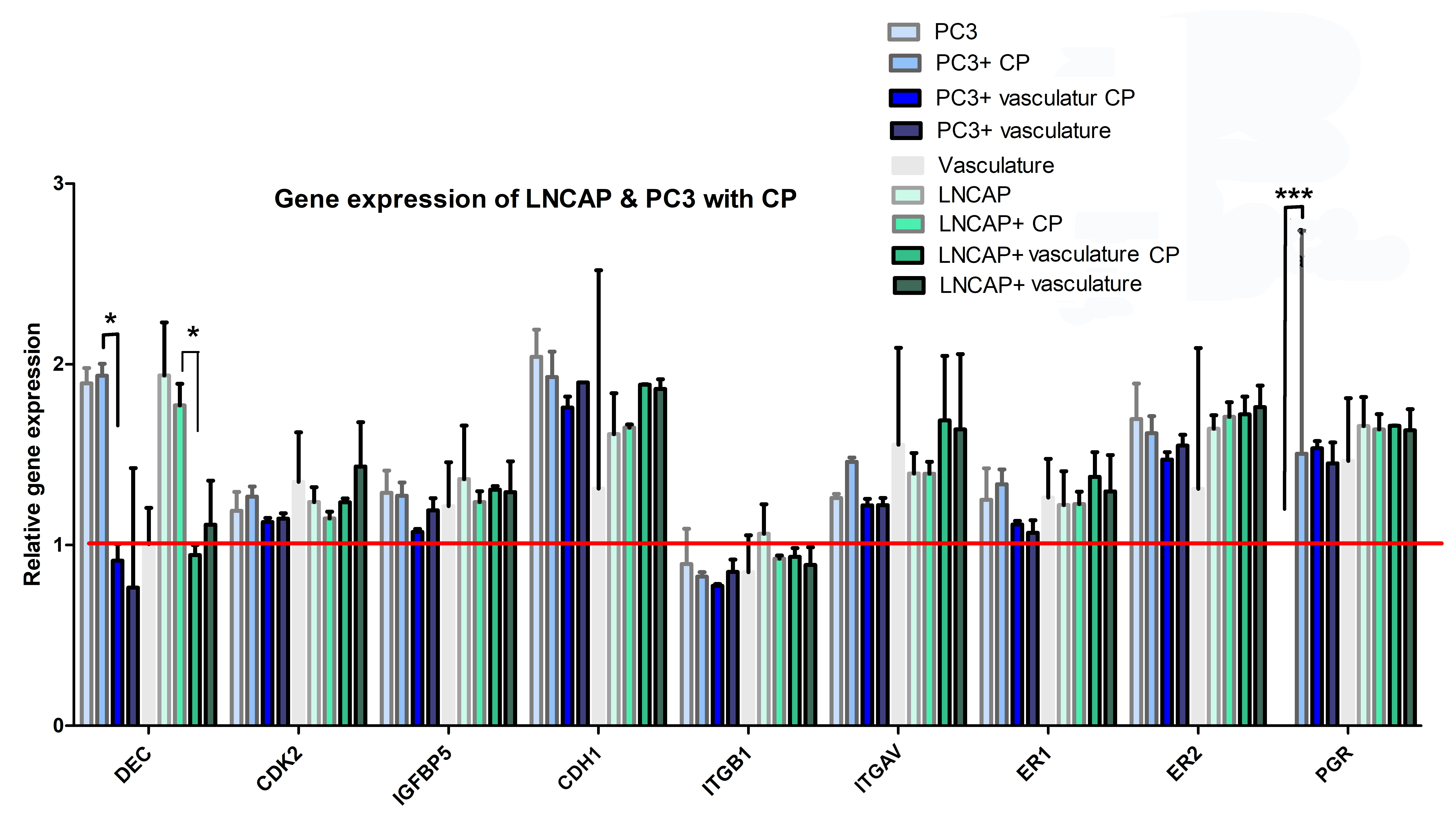

Supplement: Supplementary file 1 [file ijms-21-01833-s001.zip › ijms-723674-supplementary/Revised Supplementary files_Huttala/Figure S5_CP R.tif]
